# Host-specific Nod-factors associated with *Medicago truncatula* nodule infection differentially induce calcium influx and calcium spiking in root hairs

**DOI:** 10.1111/nph.12475

**Published:** 2013-09-10

**Authors:** Giulia Morieri, Eduardo A Martinez, Andrzej Jarynowski, Hugues Driguez, Richard Morris, Giles E D Oldroyd, J Allan Downie

**Affiliations:** 1 John Innes CentreNorwich Research Park, Colney, Norwich, NR4 7UH, UK; 2 Centre de Recherches sur les Macromolécules Végétales, CNRSB.P. 53, F-38041, Grenoble CEDEX 9, France

**Keywords:** calcium influx, ethylene, infection, *Medicago truncatula*, Nod factor, nodulation

## Abstract

- Rhizobial nodulation (Nod) factors activate both nodule morphogenesis and infection thread development during legume nodulation. Nod factors induce two different calcium responses: intra-nuclear calcium oscillations and a calcium influx at the root hair tip. Calcium oscillations activate nodule development; we wanted to test if the calcium influx is associated with infection.
- *Sinorhizobium meliloti nodL* and *nodF* mutations additively reduce infection of *Medicago truncatula*. Nod-factors made by the *nodL* mutant lack an acetyl group; mutation of *nodF* causes the nitrogen (N)-linked C16:2 acyl chain to be replaced by C18:1. We tested whether these Nod-factors differentially induced calcium influx and calcium spiking.
- The absence of the NodL-determined acetyl group greatly reduced the induction of calcium influx without affecting calcium spiking. The calcium influx was even further reduced if the N-linked C16:2 acyl group was replaced by C18:1. These additive effects on calcium influx correlate with the additive effects of mutations in *nodF* and *nodL* on legume infection. Infection thread development is inhibited by ethylene, which also inhibited Nod-factor-induced calcium influx.
- We conclude that Nod-factor perception differentially activates the two developmental pathways required for nodulation and that activation of the pathway involving the calcium influx is important for efficient infection.

## Introduction

The formation of nitrogen (N)-fixing nodules on legumes requires coordinated signalling between rhizobia and the plant. This results in nodule morphogenesis and the development of specialized tube-like infection structures (infection threads), which often initiate in root hair cells; rhizobia grow down infection threads to infect plant cells in the developing nodule (Oldroyd *et al*., 2011). In most legumes activation of these programmes requires rhizobially-produced nodulation signals. These ‘Nod factors’ are chitin oligomers carrying various different substitutions on the oligosaccharide backbone and N-linked acyl groups, which in some rhizobia such as *Sinorhizobium meliloti* and different biovars of *Rhizobium leguminosarum* have different chain lengths (C16–20) and/or degrees of saturation. These various substitutions are the major determinants of host specificity for a given rhizobial strain (Perret *et al*., 2000).

A Nod-factor-induced signalling pathway activates nodule morphogenesis (Oldroyd *et al*., 2011). Nod-factors bind with high affinity to extracellular LysM domains on two plasma-membrane receptor-kinases (NFR1 and NFR5) in the legume *Lotus japonicus* (Broghammer *et al*., 2012). This binding activates a pathway leading to calcium oscillations (called calcium spiking) in and around the nucleus (Ehrhardt *et al*., 1996). Calcium spiking is decoded by a calcium and calmodulin-binding kinase (CCaMK) and gain-of-function mutations in CCaMK activate nodule development in the absence of rhizobia and Nod-factors (Gleason *et al*., 2006; Tirichine *et al*., 2006). Nodule organogenesis, but not rhizobial infection also requires a cytokinin receptor (Murray *et al*., 2007) and gain-of-function mutations in this cytokinin receptor also induce nodule development in the absence of rhizobia (Gonzalez-Rizzo *et al*., 2006; Tirichine *et al*., 2007).

In addition to the calcium-spiking pathway activating nodule morphogenesis, Nod factors induce other responses in root hairs including root-hair deformation, the production of reactive oxygen species (ROS) (Cardenas *et al*., 2008) and the induction of a root-hair-tip-localized influx of calcium (Shaw & Long, 2003), that initiates partial membrane depolarization due to K^+^, Cl^−^ and H^+^/OH^−^ movements (Felle *et al*., 1998). Root-hair deformation and calcium influx can be separated from the nodule organogenesis pathway, since many of the genes required for activation or decoding of calcium spiking are not necessary for root hair deformation or calcium influx. However, the LysM-domain Nod-factor receptors are required for all Nod-factor responses (Radutoiu *et al*., 2003; Shaw & Long, 2003; Miwa *et al*., 2006b). The activation of calcium spiking and calcium influx can be separated because much higher concentrations of Nod-factors are required for the induction of calcium influx than calcium spiking (and root-hair deformation; Shaw & Long, 2003; Miwa *et al*., 2006b). These observations imply activation of different pathways downstream of the Nod-factor receptors, with one pathway associated with calcium spiking, one pathway associated with calcium influx and possibly another pathway activating root-hair deformation. The calcium spiking pathway regulates nodule organogenesis (Oldroyd *et al*., 2011), while the calcium influx pathway has been proposed to be associated with bacterial infection (Miwa *et al*., 2006b).

The existence of two different signalling outputs led us to question whether Nod-factor receptors can differentially activate one pathway over the other. Such discrimination may be associated with the structure of the Nod-factor molecule, since the different decorations on the Nod factors appear to differentially control nodule organogenesis and bacterial infection. For example, replacing the N-linked C16:2 acyl group on the *Sinorhizobium meliloti* Nod factor with a C18:1 group (equivalent to mutating *nodF* or *nodE*) significantly reduced rates of rhizobial infection and reduced, but did not block nodule development (Ardourel *et al*., 1994). The C18:1 modification reduces the potency of the Nod factor, making it 100 times less active for induction of calcium spiking than the C16:2 Nod factor (Oldroyd *et al*., 2001). This structural dependency on activation of the signalling pathways provides a tool for comparing the different pathways induced by the Nod-factor receptors.

In this work we assessed whether the activation of the differential signalling pathways could be discriminated based on the structure of the Nod factors. We demonstrate that removing an O-linked acetyl group from the *N*-acylated glucosamine residue had a marked effect on induction of the calcium influx without affecting calcium spiking. We also demonstrate that ethylene, which negatively regulates infection (Penmetsa & Cook, 1997), also decreases the Nod-factor-induced calcium influx. Our data support a model in which the Nod-factor receptors can discriminate between the different Nod factors to preferentially activate one modality of signalling over the other.

## Materials and Methods

### Seedling growth and calcium imaging

Seeds of *Medicago truncatula* Gaerten cv Jemalong A17 (Chabaud *et al*., 1996), *hcl1-1* (Catoira *et al*., 2001) *nin-1* (Marsh *et al*., 2007), *bit1-1* (Middleton *et al*., 2007), *nsp2-2* (Oldroyd & Long, 2003) and *skl* (Penmetsa & Cook, 1997) were germinated and grown on Fahraeus (FP) N-free plant medium (Fahraeus, 1957) agar plates containing 0.1 μM l-*α*-(2-aminoethoxy)vinyl glycine (AVG) and prepared for microscopy as previously described (Miwa *et al*., 2006a). Chambers filled with 100 μl of liquid FP medium were used to analyse roots 2–3 cm long and calcium in individual root-hairs was imaged using either injected Oregon Green 488 BAPTA-1-dextran 10 000 MW and Texas Red-dextran 10 000 MW (Molecular Probes, Eugene, OR, USA) or with yellow Cameleon YC 2.1, *M. truncatula* transgenic seedlings as described (Miwa *et al*., 2006a,2006b).

### Nod factor preparation

The *nodL* (LCO IV C_16:2_, S) and *nodF/nodL* (LCO IV C_18:1_, S) Nod factors were synthesized using ‘*E. coli* cell factory’ procedure previously described (Samain *et al*., 1999; Maillet *et al*., 2011). Wild type (WT) Nod factor was extracted from culture supernatant of *Sinorhizobium  meliloti exo7* carrying pMH682 (Honma *et al*., 1990) essentially as described (Firmin *et al*., 1993). Briefly, Nod factor from supernatant of a 2 l culture of *S. meliloti* induced with luteolin (0.5 μM) was pumped through a C18:1 reverse phase column (Sep-Pak; Waters, Elstree, UK) and then eluted with 2.5 ml of each of 20%, 40%, 60%, 80% methanol and 4 ml of 100% methanol. The fractions were assayed for root-hair deformation activity (Miwa *et al*., 2006b) and the 80% methanol fraction which had the highest activity was subsequently used. Nod factor was then analysed by co-chromatography with a standard on a reverse phase C18 high-performance liquid chromatography column (Phenomenex, Macclesfield, UK) eluted using a linear gradient of acetonitrile vs 0.1% formic acid in water. The sample was analysed using a Thermo DecaXPplus ion trap (equipped with a Surveyor HPLC system; Fisher Scientific, Loughborough, UK), collecting full mass data in negative mode using electrospray ionization. The *S. meliloti* IV C_16:2_, S, Ac Nod factor concentration in the 80% methanol extract was estimated to be 1.09 mM.

### Algorithm to analyse calcium influx

Based on the defined influx characteristics in the main text, we developed an automated geometric analysis. To reduce the risk of random dye movements biasing the results we used a moving average to capture the overall background trend and assumed that influx should be detectable in both the tip and shaft region. The area between adjacent minima of a second-order polynomial fit to the data and the trace was used to determine the large deviations we associate with a calcium influx. These areas were weighted by the local variance to reduce the scores in spiking regimes. By multiplying the influx scores for data obtained from the shaft and tip region we obtain an influx score for each cell.

## Results

### Analysis of Nod-factor-induced calcium influx responses

**Figure 1:**
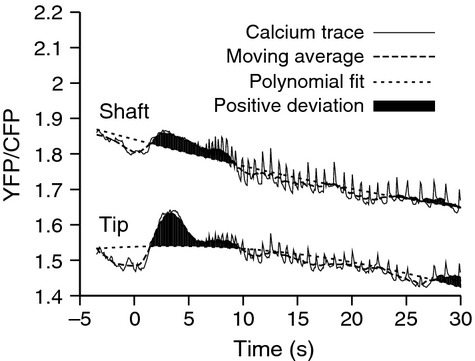
Detection of Nod factor-induced calcium influx. The calcium changes at the tip and the shaft of a single root hair on transgenic *Medicago truncatula* expressing the Cameleon YC2.1, were assayed by measuring the ratio of YFP and CFP fluorescence over time, following the addition of Nod factor at time zero. A second-order polynomial fit to the calcium trace acts as a reference baseline and a moving average approximation to the calcium trace captures the overall trend while integrating out small sharp fluctuations such as spikes and noise. The positive deviations between the polynomial fit and the moving average are shaded, and these deviations, when multiplied from the tip and shaft region are taken as a measure of the likelihood of an influx being present.

Confidently identifying a calcium influx in root hairs is difficult because it is a single transient event that can be affected by cytoplasmic streaming. To develop an objective analysis of what constitutes a calcium influx, we defined an influx by an above-average, broad variation in the background trend (Fig. 1) and made use of the observation that the Nod-factor-induced calcium influx originates at the root hair cell tip and propagates towards the base (Shaw & Long, 2003). Fluorescence images were taken of areas *c*. 10 μm from the tip of the root hair, and the region of the shaft of the root hair cell protruding from the root. Cells showing a significant calcium increase in both regions resulted in flux scores (see the Materials and Methods section) above 0.1 and were considered as candidate cells positive for calcium influx. From close inspection of many traces, we found this approach captured our definition of influx robustly with a strong correlation between the score and our own visual evaluation. Below a score of 0.1 traces were classified as negative.

**Figure 2:**
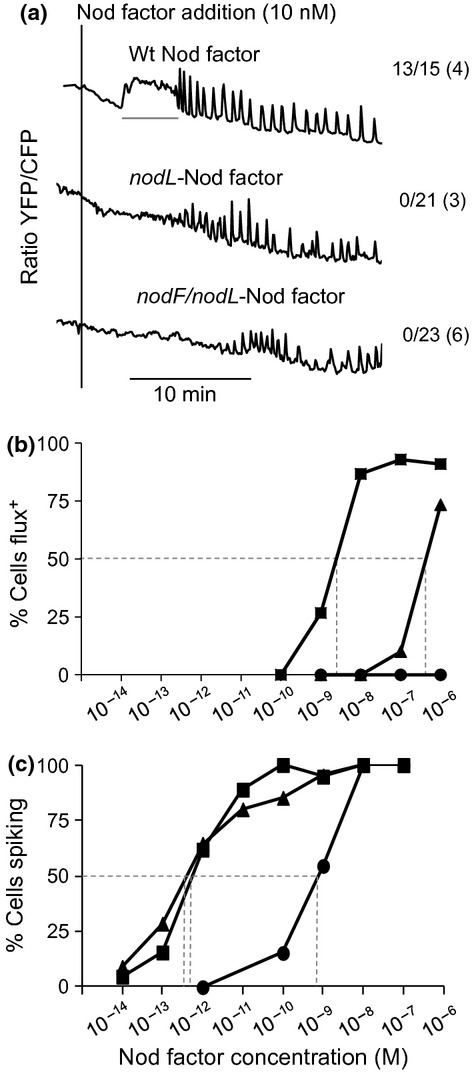
Induction of calcium influx by *nodL-* and *nodF/nodL-* Nod factors. (a) Changes of intracellular calcium in wild-type *Medicago truncatula* YC 2.1 root hairs treated with 10 nM wild type (NodSm-IV C_16:2_, S, Ac), *nodL* (LCO-IV C_16:2_, S) or *nodF/nodL* (LCO-IV C_18:1_, S) Nod factors. The traces show data of changes in fluorescence of ratio of yellow to cyan (YFP:CFP) fluorescence (arbitrary units). The vertical line indicates time of addition of Nod factor and the horizontal line shows calcium influx. The numbers indicate the numbers of cells with calcium influx/total number of cells analysed with each Nod factor and the number of plants tested is shown in parentheses. (b) Multiple cells from wild type plants were assessed for the induction of calcium influx in response to different concentrations of wild type (squares), *nodL* (triangles) and *nodF/nodL* (circle) Nod factors. The percentages of cells that showed calcium influx at each concentration are indicated. (c) As (b) except that the percentages of cells showing calcium spiking are indicated. In (b) and (c) a minimum of 10 root hair cells from at least three different plants were tested for each data point.

We tested this system by analysing calcium influx transgenic *Medicago truncatula* seedlings expressing Yellow Cameleon YC2.1 (Miwa *et al*., 2006a) following induction by 10^−6^–10^−10^ M Nod factor (Fig. 2b). The observation that the titration curve started with no cells showing calcium influx rising to nearly all cells showing calcium influx validates this method.

### *nodL-* and *nodF/nodL-* Nod factors are impaired for induction of calcium-flux

Mutation of *nodL* blocks the attachment of the O-linked acetyl group (Bloemberg *et al*., 1994) to Nod factors and causes a delay and reduction in infection (Ardourel *et al*., 1994). The effects of absence of the acetyl group (see Supporting Information Fig. S1c) were assayed using LCO-IV C_16:2_, S (referred to hereafter as *nodL*-Nod factor) added to *Medicago truncatula* seedlings expressing Yellow Cameleon YC2.1 (Miwa *et al*., 2006a) to monitor calcium. No calcium influx was observed with 10 nM *nodL*-Nod factor, although calcium spiking was induced (Fig. 2a). At 10 nM, WT Nod factor (NodSm-IV C_16:2_, S, Ac, Fig. S1a) induced calcium influx in 13 of the 15 the cells tested (Fig. 2a).

The induction of calcium influx and calcium spiking was assayed over a range of 10^−6^–10^−10^ M Nod factor. It required at least 100-fold more *nodL* Nod factor to induce the calcium influx than WT-Nod factor (Fig. 2b) with 50% of root hairs showing calcium influx at 5 × 10^−7^ M *nodL*-Nod factor, compared to 3 × 10^−9^ M for WT*-*Nod factor (Fig. 2b). By contrast, calcium spiking induced by *nodL*-Nod factor was not significantly different from the WT-Nod factor (Fig. 2c) as was seen previously with microinjected root hairs (Oldroyd *et al*., 2001). This shows that the lower activity of the *nodL*-Nod factor for induction of calcium influx is not due to an error in estimation of the Nod factor concentration. It also shows that the NodL-determined acetyl group is important for induction of calcium influx but not for calcium spiking.

The observations that the acetyl group is required for both efficient induction of calcium influx (Fig. 2) and for efficient infection thread formation (Ardourel *et al*., 1994), support the hypothesis that calcium influx may be important for infection thread formation. If this is correct we would predict that there would be even more impairment of induction of calcium influx by the Nod factor as produced by the *Sinorhizobium meliloti nodF-nodL* double mutant, which induces root-hair deformation but is almost completely blocked for infection thread formation (Ardourel *et al*., 1994). To test this we analysed the induction of calcium influx using a Nod factor which carries a C_18:1_ *N*-acyl chain (rather than the *nodF-nodE*-determined C_16:2_ or C_16:3_) and also lacks the NodL-determined acetate group (Fig. S1b). This signal (LCO-IV C_18:1_, S), equivalent to that produced by the *S. meliloti nodF,nodL* double mutant, (referred to hereafter as the *nodF/nodL*-Nod factor) did not induce a calcium influx at 10 nM (Fig. 2a) or even at 1 μM (Fig. 2b). The *nodF/nodL*-Nod factor was *c*. 1000 times less active than WT*-*Nod factor for induction of calcium spiking (Fig. 2c), as found previously (Oldroyd *et al*., 2001). These observations are consistent with the *nodF/nodL* mutant being completely blocked for infection thread and nodulation, whereas a very few infections by *nodL* mutant can eventually lead to delayed and reduced nodulation (Ardourel *et al*., 1994).

### Analysis of calcium influx in *Medicago truncatula* mutants defective for infection

**Figure 3:**
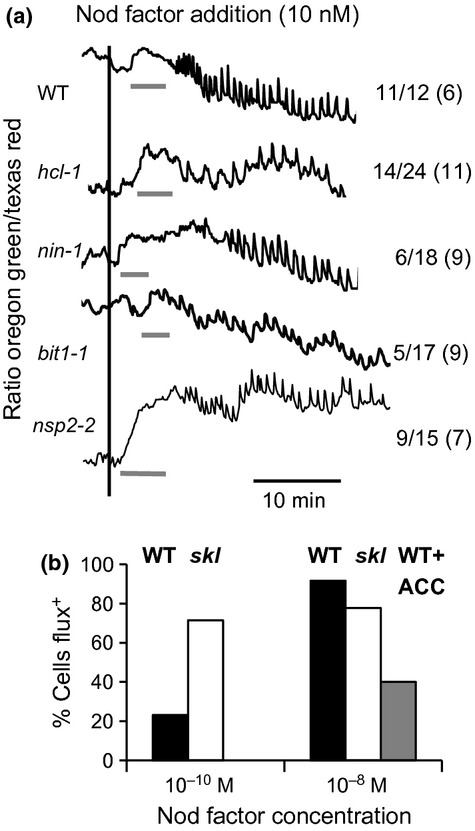
Effects of mutations affecting infection and ethylene perception on Nod-factor-induced calcium influx in *Medicago truncatula*. Nod-factor-induced calcium influx was measured in root hairs of *M. truncatula* wild-type (WT) and mutants by measuring at 5-s intervals the ratios (arbitrary units) of fluorescence of Oregon Green (calcium sensitive-dye) and Texas Red (calcium-insensitive dye). (a) Representative traces of ratios from the shafts of root hairs of WT, *hcl-1, nin-1, bit1-1*and *nsp2-2* mutants. The vertical line indicates the addition of 10 nM Nod factor and the horizontal lines indicate the regions of the traces where the algorithm predicts a calcium influx. The number of root hairs showing calcium influx relative to the total number of root hairs tested is shown; the number of plants tested is shown in parentheses. (b) Calcium influx was measured with WT (black bars) and skl mutant (white bars) after the addition of 0.1 or 10 nM Nod factor and show the percentage of root hair cells scoring positive for the calcium influx. At least 10 root hair cells from at least three different plants were assessed for each assay. The difference between wild type plants after 0.1 and 10 nM Nod factor was significant (*P *<* *0.001) as was the difference between wild type and *skl* plants at 0.1 nM Nod factor (*P *<* *0.02) but there was no significant difference between the WT and *skl* mutant at 10 nM Nod factor or between the *skl* mutant treated with 0.1 and 10 nM Nod factor. Treatment of WT with 10 μM ACC for 1 h before addition of Nod factor significantly (*P *<* *0.01) inhibited calcium influx (grey bar). Significance was determined using a Chi squared test.

The *dmi1*/*pollux*, *dmi2*/*symrk*, *castor*, *nup85*, *nup133, nena* and *cyclops* nodulation signalling mutants are all defective for calcium spiking but retain calcium influx (Shaw & Long, 2003; Miwa *et al*., 2006b; Groth *et al*., 2010). However, genes required for calcium influx but not for calcium spiking have not been identified. We measured calcium influx in four mutants of *Medicago truncatula, hcl-1, nin-1, bit1-1* and *nsp2-2* that retain calcium spiking but lack normal infection threads. The *hcl-1* mutation inactivates LYK3, the proposed Nod-factor receptor required for infection thread growth (Smit *et al*., 2007). The *nin-1* transcription-factor mutation causes increased root hair deformation, reduced infection and a failure to form nodules (Marsh *et al*., 2007). The *bit1-1* mutation affects the ERN-1 transcription factor required for nodulation and the initiation of infection threads (Middleton *et al*., 2007) and the *nsp2-2* mutation affecting a GRAS domain transcription factor, causes defects in infection and cortical cell division following inoculation with *Sinorhizobium meliloti* (Oldroyd & Long, 2003). All four mutants induced calcium influx in at least some of the cells tested (Fig. 3a). Since NIN, ERN and NSP2 are all transcription factors required for infection, it is likely that they would act downstream of the calcium signalling events. As observed previously (Wais *et al*., 2000; Oldroyd & Long, 2003; Marsh *et al*., 2007), calcium spiking was normal in these mutants (Fig. 3a). LYK3, a predicted Nod-factor receptor, could have been required for the calcium influx but apparently is not, based on the induction of calcium influx in some cells of the *hcl-1* mutant (Fig. 3a). The *hcl-1* allele causes a G > E change in the conserved kinase domain (Smit *et al*., 2007), so possibly some residual function of the protein could be retained allowing calcium influx under the assay conditions. Additionally there seems to be redundancy because, in the *M. truncatula hcl* mutant, calcium spiking is normal (Wais *et al*., 2000 and Fig. 3a), whereas in *Lotus japonicus,* calcium spiking and influx are blocked by mutation of *NFR1* (the possible *LYK3* orthologue; Miwa *et al*., 2006b).

### Ethylene causes decreased sensitivity to Nod-factor-induced calcium influx

The *Medicago truncatula skl* mutant, which is insensitive to ethylene, forms ten times more nodules and many more infection threads than WT (Penmetsa & Cook, 1997). If calcium influx is required for infection thread formation we would predict that the *skl* mutant should induce a calcium influx at lower concentrations of Nod-factor than seen with WT. At 0.1 nM WT-Nod factor, significantly more *skl* mutant root hairs showed induction of calcium influx than seen with WT (Fig. 3b). At 10 nM Nod-factor, this differential was lost (Fig. 3b), indicating that the *skl* mutant is more sensitive to Nod factor for the induction of calcium influx and implies that endogenous ethylene may suppress the Nod-factor-induced calcium influx.

We added the ethylene precursor, 1-amino-cyclo-propane-carboxylic acid (ACC), to WT roots and found that it significantly reduced the number of root-hair cells inducing a calcium influx after the addition of 10 nM WT-Nod factor (Fig. 3b) confirming that ethylene suppresses the calcium influx. Taken together these results show that ethylene inhibits both infection thread growth and calcium influx.

## Discussion

The observations that the *nodL*-Nod factor induces normal calcium spiking but is much less potent than WT-Nod factor for the induction of the calcium influx and both these responses require NFP (Wais *et al*., 2000; Amor *et al*., 2003; Shaw & Long, 2003; Miwa *et al*., 2006b) imply that Nod factor perception via NFP must have two different outputs, one leading to calcium spiking and the other leading to calcium influx.

We know little about what is involved in this calcium influx pathway during legume infection, but since infection thread initiation can be considered to be an extension of root-hair tip growth (albeit directed backwards into the cell), these two forms of polar growth may have some similar properties. Root-hair tip elongation requires NADPH-oxidase driven production of ROS, activated by small ROP-family GTPases which can bind to and activate NADPH oxidase (Wong *et al*., 2007). The production of ROS activates an inflow of calcium at the root-hair tip (Foreman *et al*., 2003), causing a positive feedback on NADPH activity (Takeda *et al*., 2008). This pathway regulates the changes in cytoskeletal dynamics, vesicular trafficking and signalling lipids associated with regulation of apical growth (Yang, 2008).

**Figure 4:**
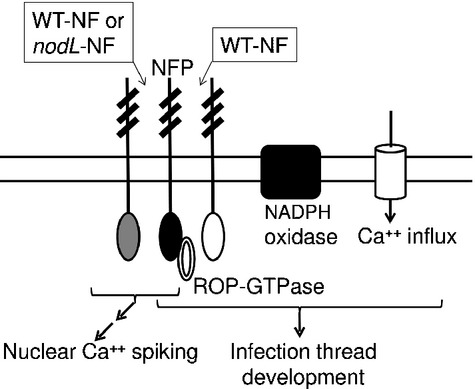
Model of activation of both nodule morphogenesis and infection thread development following activation of Nod-factor receptors. NFP and related LysM-domain receptors are represented showing the three extracellular LysM domains and the intracellular kinase-like domains. Calcium spiking is induced by similar concentrations of wild-type (WT) and *nodL*-Nod factors, whereas induction of the calcium influx requires 100-fold more *nodL*-Nod factor than WT-Nod factor. It is speculated that the calcium influx which may play a role in infection is activated by a pathway that involves (1) a receptor complex analogous to that formed between NFR1 and NFR5 in *Lotus japonicus* (Madsen *et al*., 2011), (2) a ROP-GTPase that binds to NFP (NFR5) analogous to that identified by Ke *et al*. (2012) and (3) the production of reactive oxygen species (ROS) by activation of a root-hair NADPH oxidase analogous to that identified by Montiel *et al*. (2013)

There are parallels with nodulation signalling associated with infection: (1) the calcium influx, membrane depolarization and production of ROS are induced by similar concentrations of Nod factor (Oldroyd & Downie, 2004); (2) the Nod-factor receptor NFR5 binds to the small GTPase ROP6, which is involved in infection-thread growth (Ke *et al*., 2012), and (3) an NADPH oxidase is required for rhizobial infection of *Phaseolus* bean (Montiel *et al*., 2013). Our observation that the *nodL*-Nod factor is greatly reduced for induction of the calcium influx (but not calcium spiking) fits with a model in which Nod-factor binding normally activates two pathways, one being calcium spiking and the other involving NADPH-oxidase and the calcium influx. It is proposed that this latter pathway could help activate infection by rhizobia (Fig. 4).

How could Nod-factor binding to cognate receptors induce both calcium-spiking-driven nodulation development and the proposed NADPH-oxidase-calcium influx-dependent infection pathway? The *Medicago truncatula* Nod-factor receptor NFP and its *Lotus japonicus* orthologue NFR5 are essential for both responses. One way of achieving two outputs could be to have Nod-factor-induced cooperative interactions between NFP and itself (or another receptor), similar to that reported for chitin binding to the Arabidopsis CERK1 receptor (Liu *et al*., 2012), such that the resulting interaction alters the kinase activity or specificity of the receptor complex. The interaction between the two LysM-domain receptors NFR1 and NFR5 (Madsen *et al*., 2011) suggests that this is likely. Such interactions could result in differential activation of downstream signalling components especially taking into account that one output could be mediated directly by the kinase activity of the receptor and another could be mediated via an activation of a ROP-GTPase (Fig. 4). Different outputs from Nod-factor receptors have been proposed previously (Miwa *et al*., 2006b; Hayashi *et al*., 2010; Madsen *et al*., 2010), and this model could explain how such different outputs could be achieved. We thought it possible that *HCL* encoding the LYK3 receptor might play such a role, but the *hcl* mutant retained the Nod-factor-induced calcium influx so the Nod-factor-induced calcium influx observed in the *hcl-1* mutant could be explained by an interaction between NFP and one or more of the other predicted receptors in this family.

Ethylene reduces the sensitivity of legumes to Nod factor based on analyses of calcium spiking and gene induction (Oldroyd *et al*., 2001). The *Medicago truncatula skl* mutant is defective in ethylene signalling due to a mutation in the *EIN2* gene and infection threads in this mutant are more prolific and grew in an uncontrolled fashion throughout the cortex (Penmetsa & Cook, 1997; Murray *et al*., 2007). The observation that the *skl* mutant was more sensitive to Nod factor for induction of calcium influx, together with the observation that the ethylene precursor, ACC, inhibited induction of calcium influx shows that ethylene can suppress the calcium influx and in this way may regulate infection. The combined observations of the effect of ethylene and the different Nod-factor structures on the activation of calcium influx and promotion of bacterial infection, all point towards a role for the calcium influx in the initiation and/or proliferation of bacterial infection.

## Supporting Information

**Fig S1** Structures of Nod factors made by WT, *nodL* and *nodF/nodL* mutants of *S. meliloti*.
