# Supplementary material for: Host-specific Nod-factors associated with Medicago truncatula nodule infection differentially induce calcium influx and calcium spiking in root hairs
Source: New Phytol. 2013 Sep 10;200(3):656–62. doi: 10.1111/nph.12475 (PMC3908372; doi:10.1111/nph.12475)
Supplement: Supplementary file 1 — Fig S1 Structures of Nod factors made by WT, nodL and nodF/nodL mutants of S. meliloti. [file nph0200-0656-sd1.pdf]

(a) **Wild type Nod factor**

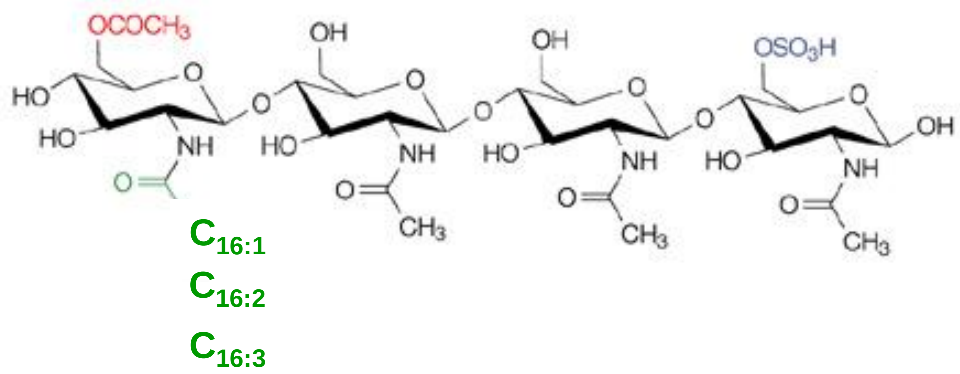

(b) ***nodF/nodL* Nod factor**

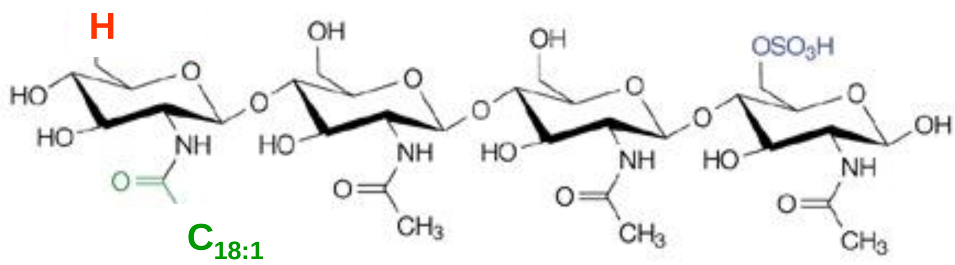

(c) ***nodL* Nod factor**

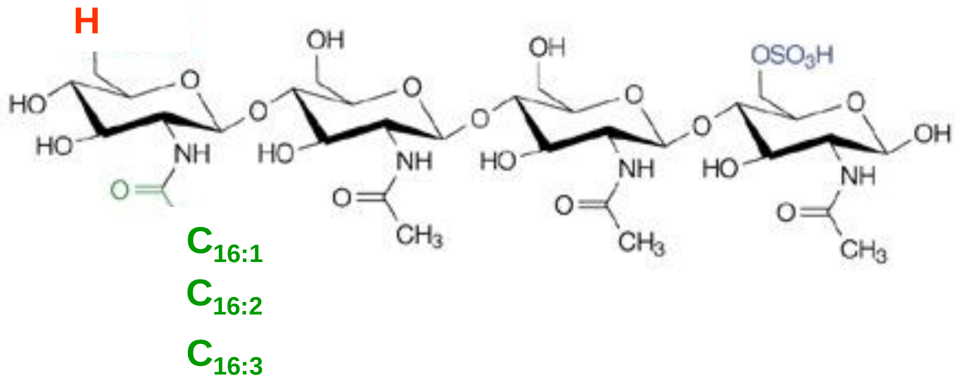

Supporting Information Fig. S1 Structures of Nod factors made by WT, *nodF/nodL* or *nodL* or mutants of *S. meliloti*

The WT Nod factors (a) are oligomers of 4 or 5 (not shown) β-1-4-linked *N*-acetyl glucosamines carrying O-linked acetyl (red) and sulfate (blue) groups and $C_{16:1}$, $C_{16:2}$ or$C_{16:3}$ N-linked acyl groups (green). Mutation of nodL (b and c) blocks attachment of the acetyl group and mutation of nodF (b) results in Nod factors carrying a $C_{18:1}$ (rather than $C_{16:1}$, $C_{16:2}$ or $C_{16:3}$) linked acyl group (green)
